# Neural Correlates of Extraversion and Trait Creativity: A Graph Theory-Based Whole-Brain Functional Network Modularity Analysis

**DOI:** 10.3390/jintelligence14060094

**Published:** 2026-06-01

**Authors:** Xiaoqian Ding, Fabin Dai, Xingbang Gai, Yi-Yuan Tang

**Affiliations:** 1 College of Psychology, Liaoning Normal University, Dalian 116029, China; ye_white@foxmail.com (F.D.);; 2 College of Health Solutions, Arizona State University, Phoenix, AZ 85004, USA

**Keywords:** trait creativity, extraversion, modularity, graph theory, brain network, resting-state fMRI

## Abstract

Background: Trait creativity is linked to brain functional connectivity, but prior studies have focused on isolated networks, neglecting whole-brain architecture. Extraversion overlaps with creativity, yet its neural mechanisms remain unclear. Objective: Our objective was to investigate whether whole-brain functional network modularity (Q) mediates the relationship between extraversion and trait creativity. Methods: Forty-three healthy university students underwent resting-state fMRI and completed extraversion and creativity scales. Whole-brain functional networks were constructed using the AAL atlas. Modularity (Q) was computed from binary networks. Bivariate correlations and mediation analysis were performed. Results: Extraversion correlated positively with creativity (r = 0.38, *p* = .011) and modularity (r = 0.37, *p* = .014). Modularity correlated positively with creativity (r = 0.41, *p* = .007). Mediation analysis revealed a significant indirect effect of extraversion on creativity through modularity (ab = 0.113, 95% CI [0.005, 0.275]), with a non-significant direct effect. Conclusions: Whole-brain network modularity statistically mediates the extraversion–creativity relationship. Higher extraversion is associated with increased modularity, which in turn is associated with higher creativity. These findings provide preliminary associative evidence for a brain network basis linking personality to trait creativity. The results reflect cross-sectional statistical patterns and require replication in larger, longitudinal samples.

## 1. Introduction

Creativity is one of the most highly valued human qualities (Sen & Hagtvet, 1993). Creativity is generally defined as the ability to generate ideas that are both original and effective (Runco & Jaeger, 2012). Research has shown that creativity exerts a profound influence across multiple domains, such as science, art, and business, thereby affecting both individual and societal progress and development (Ivcevic, 2007; Qu et al., 2015, 2017; Schmidt, 2010). Furthermore, research shows that individuals with creative personality traits such as imagination, curiosity, courage to take on challenges, and willingness to explore are more likely to make scientific discoveries (W. Li et al., 2015). Therefore, investigating trait creativity and its influencing factors holds significant academic and practical importance (Tang et al., 2024).

The Williams Creativity Scale (WCS) assesses an individual’s dispositional creativity, which comprises four dimensions: adventure, curiosity, imagination, and challenge (Wang & Wu, 2023). Individuals with high dispositional creativity are more willing to try new things and accept the possibility of failure, hold stronger interest and desire to explore new knowledge and experiences, and are more ready to take on challenges and strive for solutions when facing complex and difficult problems (Ackerman, 2017; Henriksen et al., 2021).

Interestingly, traits similar to creative personality have been found in highly extraverted individuals (McCrae & John, 1992). Highly extraverted individuals typically exhibit behavioral characteristics such as being energetic and socially active, tend to obtain novel inspiration through interpersonal interaction (van Allen et al., 2021), and simultaneously possess a strong tendency towards novelty-seeking and adventure, demonstrating unique advantages in complex cognitive activities such as task switching and multi-tasking (Kinney, 2007). This overlap at the trait level suggests a significant correlation between the two (Amin et al., 2020; Furnham et al., 2013; Goclowska et al., 2019; Grajzel et al., 2023; Guo et al., 2017; Puryear et al., 2017; Yao & Li, 2021).

It is noteworthy that research on the personality correlates of creativity has long suffered from dimensional bias. Previous studies have predominantly focused on the association between the psychoticism dimension and creativity (Rawlings, 1985; Acar & Runco, 2012; Muhammad et al., 2021), while relatively neglecting extraversion, a key factor equally supported by theory. More importantly, regardless of the personality dimension under investigation, existing research has primarily relied on behavioral observation and questionnaire measurement. Few studies have delved deeply into the underlying neural basis through which personality traits influence creativity. Direct empirical support for extraversion, trait creativity, and their underlying neural mechanisms remains lacking.

Numerous studies have investigated the neural mechanisms underlying creativity. Research has demonstrated that functional connectivity within brain networks can predict an individual’s creative ability (Beaty et al., 2018). Furthermore, other studies have explored the relationship between dispositional creativity and the functional connection efficiency of specific local brain networks—namely, the default mode network (DMN) and executive network (EN). These studies have found a positive correlation between dispositional creativity and specific regional areas, including the precuneus, posterior cingulate cortex, and dorsomedial prefrontal cortex, as well as the overall integrity of the default mode network (Adnan et al., 2019; Beaty et al., 2015; Jiao et al., 2017; Kenett et al., 2020).

However, complex personality traits like trait creativity and extraversion have more intricate physiological bases. While the aforementioned studies on local brain networks provide valuable insights into the neural basis of trait creativity, they primarily focus on single networks or individual regions, overlooking the complex connectivity of the brain as a whole. Focusing solely on the activity of a single network may neglect the interactions between these networks (Hearne et al., 2016; Power et al., 2011). In contrast, graph theory analytical methods can provide more comprehensive information compared to single-network analysis, revealing complex patterns of functional connectivity between different brain regions (Liu et al., 2017; Sporns, 2018; van Wijk et al., 2010). Graph theory is a branch of mathematics that studies graph structures and their properties; graphs consist of nodes and edges (Arul et al., 2023). Modularity is a method within graph theory used to analyze and identify community structure in networks (Meunier et al., 2010). Modularity identifies groups of nodes that are more densely connected among themselves by measuring the density of connections between nodes in the graph; these node groups are called modules or communities (Meunier et al., 2010). A high modularity value indicates a clear community structure within the network, where connections between nodes within the same community are denser, while connections between communities are sparser (Newman, 2006). A high degree of modular organization allows each module to operate relatively independently and process specific types of information. This independence permits modules to function autonomously to some extent, without relying on the state of the entire network (Cowan & Jonard, 2023). Connections within modules are very dense, whereas connections between modules are relatively sparse (Sporns & Betzel, 2016). This characteristic of high internal cohesion and low external coupling facilitates efficient information transfer within modules while reducing interference (Kovács & Palla, 2021).

Numerous previous studies have demonstrated that the brain’s modularity is highly correlated with trait creativity (Y. Li et al., 2021; Ovando-Tellez et al., 2022; Cosgrove et al., 2023). The independence of modules may allow different thoughts to be processed in relatively autonomous environments, potentially encouraging the generation of diverse thinking styles. Efficiency may contribute to the rapid integration and application of information, thereby providing a favorable environment for innovation. Finally, the relative stability of modular structures may support the sustained development of thought, reducing the impact of external interference. Previous research has found that individuals high in psychoticism lack coordination in the reorganization of functional brain communities; the disordered transfer of single brain regions disrupts the originally clear network modular structure (Sassenberg et al., 2024). In contrast, extraversion levels are positively correlated with the flexibility of modular reorganization in individuals’ brain networks (Kabbara et al., 2020), thereby helping highly extraverted individuals efficiently process and integrate information in diverse social contexts (Soler-Pastor et al., 2023). Synthesizing the above research, although previous studies have separately examined the association between extraversion and brain networks (e.g., Kabbara et al., 2020) and the link between brain networks and creativity (e.g., Beaty et al., 2018), to our knowledge, limited research has integrated these three constructs within a unified mediation model. The present study seeks to address this gap by examining the interrelationships among extraversion, trait creativity, and whole-brain network modularity, with the aim of testing whether modularity may act as a statistical bridge linking extraversion and trait creativity. This approach may provide a more integrated framework for understanding the neural correlates underlying the association between personality and trait creativity. This study further explores the specific statistical associations among extraversion, trait creativity, and brain network modularity. Based on existing empirical evidence, we hypothesize that extraversion is positively associated with the modularity index, that trait creativity is positively associated with the modularity index, and that modularity may statistically mediate the association between extraversion and trait creativity.

The present study specifically focused on extraversion for two main reasons. First, extraversion is closely associated with social engagement and dynamic interpersonal interactions, which are theoretically linked to the mechanisms of brain network reorganization and modularity. Second, previous research has largely centered on other personality traits, such as openness to experience (Boldt et al., 2026) and the relationship between neuroticism and creativity (Koivisto & Toivanen, 2026). In contrast, studies on the neural pathway through which extraversion is associated with creativity via whole-brain modularity remain relatively scarce. Therefore, this study prioritized the investigation of extraversion, aiming to enrich research on the impact of different personality dispositions on creativity.

## 2. Materials and Methods

### 2.1. Participants

This study recruited 43 undergraduate students from various universities in Liaoning Province, China. Inclusion criteria were as follows: (1) self-reported good physical health with no physical illnesses; (2) no personal history or family history of mental health disorders; (3) no substance addiction and no use of any medication affecting brain excitability within three days prior to the experiment. All 43 participants (29 males) completed the experiment, with a mean age of 20.91 ± 1.72 years. Each participant volunteered, provided written informed consent before the experiment, and received compensation upon completion. The study followed the principles of the Declaration of Helsinki. This study involved human participants and was reviewed and approved by the Ethics Committee of the researcher’s university. Our protocol approval number is LL2025069.

### 2.2. Measurement Questionnaires

#### 2.2.1. Eysenck Personality Questionnaire

This study employed the extraversion subscale from the Eysenck Personality Questionnaire-Revised Short Scale for Chinese (EPQ-RSC), using a 4-point Likert scale (1 = completely disagree, 4 = completely agree), with higher scores indicating stronger extraversion (Eysenck & Eysenck, 1991; Qian et al., 2000). As the EPQ extraversion scale is unidimensional and related to social activity, it allows for such disaggregation in our study (Rocklin & Revelle, 1981). The original items included both positively and negatively worded questions; data processing unified the direction. Extraversion scores were obtained by calculating the mean of the 12 items, and all scores were converted to standard scores. This scale has been demonstrated by multiple studies to be reliable and valid for Chinese participants (Wei et al., 2011).

#### 2.2.2. The Williams Creativity Scale

The Williams Creativity Scale (Chinese version) assesses the following four dimensions: Curiosity (i.e., the tendency to explore or play with ideas; including items 2, 8, 11, 12R, 19, 27, 33, 34, 37, 38, 39, 47, 48R, and 49); Challenge (i.e., the tendency to seek new alternatives and solutions to problems, and to restore order from chaos; including items 3, 4R, 7, 9R, 10, 15, 17R, 18, 26, 41, 42, and 50); Adventure (i.e., the tendency to act under uncertain conditions and defend one’s own views; including items 1, 5, 21, 24, 25, 28, 29R, 35R, 36, 43, and 44); and Imagination (i.e., the tendency to imagine and construct mental images or intuitive perceptions; including items 6, 13, 14, 16, 20, 22, 23, 30, 31, 32, 40, 45R, and 46). “R” denotes reverse-scored items. Participants responded on a 3-point Likert scale, ranging from 1 (strongly disagree) to 3 (strongly agree). The Chinese version of this scale demonstrated good reliability and validity, with a Cronbach’s α of 0.88, and its construct validity was supported by confirmatory factor analysis (C.-P. Li et al., 2023).

### 2.3. Resting-State fMRI Data Acquisition

**Figure 1:**
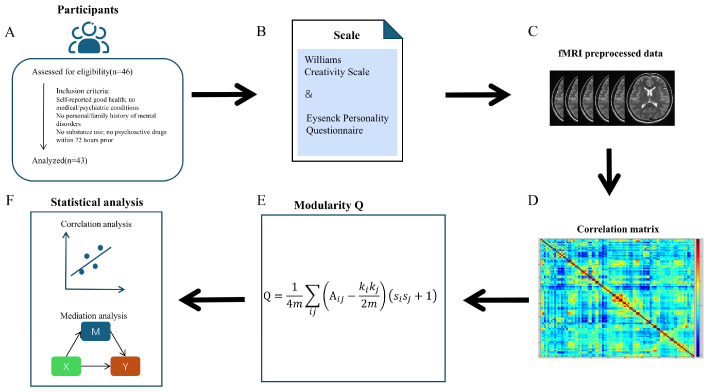
Study procedure. (**A**) Participants were screened and enrolled based on inclusion criteria: self-reported good physical health, absence of physical and mental illnesses, no personal or family history of mental disorders, no substance abuse, and no intake of psychoactive drugs within 72 h before the experiment. (**B**) Participants completed the WCS and Eysenck Personality Questionnaire. (**C**) Resting-state scanning data were collected. (**D**) fMRI signals were preprocessed to generate functional connectivity heatmaps. (**E**) Modularity index Q was calculated. (**F**) Mediation analysis was performed.

All brain imaging data were acquired using a 3.0 Tesla Siemens scanner. Functional images were collected using an echo-planar imaging sequence with 36 slices (repetition time = 2000 ms, echo time = 30 ms, flip angle = 80°, field of view = 23 cm, matrix = 64 × 64, slice thickness = 4 mm, no gap). Structural imaging was performed using a magnetization-prepared rapid gradient-echo sequence (repetition time = 7 ms, echo time = 3.2 ms, flip angle = 8°, matrix = 256 × 256, slice thickness = 1 mm). During the 6 min scan, participants were instructed to fixate on a small white crosshair at the center of a black screen and remain still. Some studies suggest that this resting-state pattern differs from eyes-closed rest only in auditory networks, with no differences in default mode, attention, visual, and motor networks. Following previous paradigms, participants were told to relax during the scan, with the purpose of looking at the crosshair being to maintain proper head orientation without requiring excessive attention. The study procedure is illustrated in Figure 1.

### 2.4. Resting-State fMRI Data Processing and Calculation

Image preprocessing and further analysis were performed using SPM8 (www.fil.ion.ucl.ac.uk/spm (accessed on 14 February 2025)) and the Resting-State fMRI Data Analysis Toolkit (http://resting-fmri.sourceforge.net (accessed on 27 March 2025)). To ensure magnet stabilization, the first 10 volumes were discarded. The 35th slice was used as a reference for slice timing correction. Head motion correction was performed. Subsequently, T1 images were segmented and co-registered with functional images to the Montreal Neurological Institute space, resampled to 3 × 3 × 3 mm^3^ voxels. Spatial smoothing was applied using a Gaussian kernel with a full-width at half-maximum of 6 mm, and detrending was performed to reduce noise. Additionally, nuisance signals were removed from the data through linear regression (white matter, cerebrospinal fluid, and Friston 24 parameters). fMRI data were then temporally band-pass filtered (0.01–0.08 Hz). This study used the Automated Anatomical Labeling (AAL) atlas with 90 brain regions to parcellate the whole brain (Rolls et al., 2020). To quantify statistical dependencies between brain regions, a 90 × 90 functional connectivity matrix was constructed for each participant, where each element represented the Pearson correlation coefficient between the time series of a pair of AAL regions. Correlation coefficients were transformed using Fisher’s r-to-z transformation to improve normality. To effectively remove weak functional connections and noise, and to highlight the core functional connection structure of the network, we used GraphVar 2.03a software (https://www.nitrc.org/projects/graphvar (accessed on 30 March 2025)) to compute undirected binary networks at a relative network threshold of 0.3 (Ding et al., 2026). Modularity was calculated at a relative threshold of 0.3 to highlight core network structure. Future studies may test multiple thresholds (0.2–0.35) to examine result robustness.

Modularity (Q) is a metric in network science used to measure the quality of network partitions. Modularity quantifies the difference between the density of connections between nodes in an actual network and the expected connection density in a random network. Suppose our network contains *n* vertices. For a specific partition dividing the network into two groups, let s_i_ = 1 if vertex i belongs to group 1, and s_i_ = −1 if it belongs to group 2. Let A_ij_ represent the number of edges between vertices i and j. Meanwhile, if edges are placed randomly, the expected number of edges between vertices i and j would be k_i_k_j_/2*m*, where k_i_ and k_j_ are the degrees of the vertices, and m = ½∑*_i_k_i_* is the total number of edges in the network. Therefore, modularity Q is given by the sum of A_ij_ − k_i_k_j_/2m over all pairs of vertices i, j that belong to the same group. Noting that the quantity ½(*s_i_s_j_*+ 1) is 1 if i and j are in the same group and 0 otherwise, modularity can be expressed as $Q=\frac{1}{4m}\sum_{ij}\left(A_{ij}-\frac{k_{i}k_{j}}{2m}\right)\left(s_{i}s_{j}+1\right)$ (Newman, 2006).

### 2.5. Correlation and Mediation Analysis

To further investigate the relationships among extraversion, WCS, and Q, bivariate correlations between these three variables were calculated. Calculations were performed in SPSS 19 (SPSS Inc., Chicago, IL, USA). Additionally, to examine whether Q could mediate the relationship between extraversion and WCS, mediation analysis was conducted using PROCESS (an SPSS toolbox, www.processmacro.org/index.html (accessed on 31 March 2025)). All variables were standardized into Z-scores prior to mediation analysis. Since standardization is a linear transformation that does not alter the distribution pattern of the original data, additional normality tests on the standardized Z-scores were not required. In constructing the mediation model, X represented extraversion, Y represented WCS, and M represented the modularity index (Q). Five thousand bootstrap samples and bias-corrected confidence intervals were reported.

## 3. Results

### 3.1. Statistical Hypothesis Testing

The absolute skewness values of all variables ranged from 0.159 to 0.351, and the kurtosis values ranged from 2.417 to 3.034, all falling within the acceptable range (|skewness| < 1, kurtosis = 2~4), indicating that the data approximately conformed to a normal distribution (see Table 1). To prevent outliers from interfering with the statistical results, the ±3 standard deviation (±3 SD) method was adopted to detect outliers. All observed values of each variable were within the ±3 SD range, and no outliers were found in the present study. Pearson product-moment correlation coefficient (r) and variance inflation factor (VIF) were used for collinearity diagnosis. The calculation formula of VIF is VIF = 1/(1 − r^2^). The Pearson correlation coefficient between the independent variable Extraversion and the mediating variable Modularity was 0.3067, showing a low positive correlation. The VIF value was 1.1038, which was lower than the empirical threshold of 5. There was no significant multicollinearity among the predictive variables (Extraversion and Modularity) in the mediation model. The variables maintained good independence, and the estimation of regression coefficients was reliable. These results support the normality and independence of key variables for subsequent correlation and mediation analyses.

### 3.2. Correlations Among Extraversion, Trait Creativity, and Modularity

**Figure 2:**
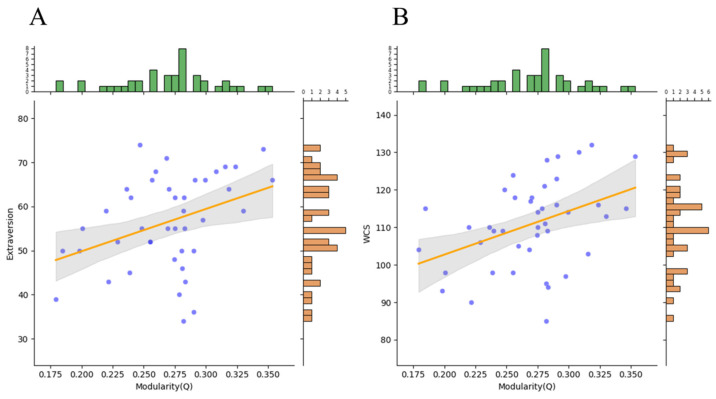
Relationship between modularity index and extraversion (**A**), and between modularity index and WCS (**B**). Purple dots represent data from each participant, and the yellow line represents the fitted line (shaded area represents 95% confidence interval). Green bars in (**A**,**B**) illustrate the distribution of the modularity index. Orange bars in (**A**) represent the distribution of extraversion scores, and orange bars in (**B**) represent the distribution of WCS scores (note: WCS = Williams Creativity Scale).

As shown in Table 2, correlation analysis revealed a positive correlation between extraversion scores and Williams Creativity Scale (WCS) scores (*r* = 0.38, *p* = .011), indicating that participants with stronger extraversion were more likely to possess higher trait creativity. To assess the feasibility of constructing a mediation model, we examined correlations among extraversion, WCS, and the modularity index. As shown in Figure 2 and Table 1, the modularity index was positively correlated with extraversion (*r* = 0.371, *p* = .014), and the modularity index was also positively correlated with WCS (*r* = 0.405, *p* = .007).

### 3.3. Mediation Analysis

**Figure 3:**
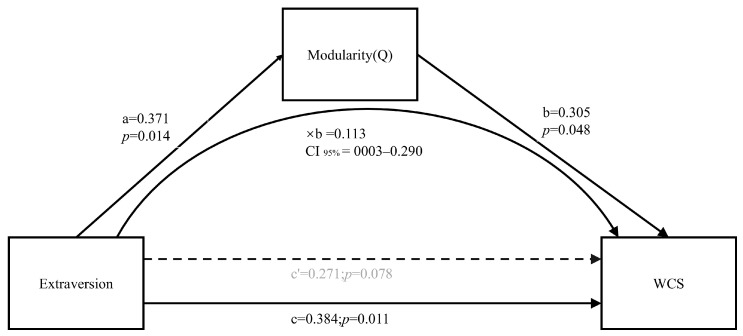
Mediation model: Modularity (Q) mediates the effect of extraversion on Williams Creativity Scale (WCS) scores. Path coefficients are shown.

As described above, extraversion, trait creativity, and the modularity index were all significantly correlated with each other. Therefore, extraversion was entered as the independent variable, trait creativity as the dependent variable, and the modularity index as the mediator in the mediation model. As shown in Table 3 and Figure 3, extraversion positively predicted the modularity index (*a* = 0.371, *p* = 0.014), and the modularity index positively predicted WCS scores (*b* = 0.305, *p* = 0.048). Although the direct effect of extraversion on WCS showed a positive trend, it did not reach statistical significance (*c*′ = 0.271, *p* = 0.079). However, the total effect of the mediation model was significant (*c* = 0.384, *p* = 0.011), and the indirect effect of extraversion on WCS through the modularity index was significant (*ab* = 0.113, 95% CI [0.003, 0.290]).

For the completeness of the analysis, the other two dimensions of the Eysenck Personality Questionnaire were subjected to the same analytical procedure. The results are presented in the Supplementary Materials (Table S1), and no significant findings were observed. Supplementary mediation analyses for neuroticism and psychoticism showed no significant indirect effects (all 95% CI included zero), indicating the observed statistical mediation is specific to extraversion.

## 4. Discussion

In this study, we examined the statistical mediating role of the brain network modularity index (Q) in the association between extraversion and trait creativity. First, we observed a significant positive association between extraversion and trait creativity. Second, the modularity index (Q) showed significant positive associations with both extraversion and trait creativity. Mediation analysis indicated that modularity (Q) showed a cross-sectional statistical mediation pattern linking extraversion to trait creativity. The indirect association was statistically significant, while the direct effect was not. These results should be interpreted as statistical associations rather than causal relationships. Taken together, these results are consistent with a full mediation model in which the modularity index statistically accounts for the link between extraversion and trait creativity.

Consistent with previous research, we also found a significant positive correlation between extraversion and trait creativity (Mammadov, 2022; Sen & Hagtvet, 1993). This result may be attributable to several factors. First, extraverted individuals tend to engage in social interaction and environmental exploration, providing opportunities to encounter diverse perspectives, thereby helping them gain more inspiration to enhance trait creativity (Soler-Pastor et al., 2023). Second, extraversion is closely associated with positive affect and motivation (Verduyn & Brans, 2012), and positive affect can enhance creative thinking (Langley, 2018). By exhibiting higher extraversion, individuals are more likely to experience positive emotional states, thus promoting trait creativity. Finally, highly extraverted individuals are more willing to embrace risk-taking or novelty, traits that can help them enhance their creative problem-solving abilities (McCrae, 1987; Zuckerman & Kuhlman, 2000). Individuals with higher extraversion possess higher levels of trait creativity. Cultivating individuals’ extraversion could potentially enhance their trait creativity. However, some studies have suggested a nonlinear relationship between the two: moderate extraversion exerts the most prominent facilitative effect on creativity, whereas extreme extraversion may conversely inhibit it (Shaw & Yu, 2023). Such inconsistencies in research findings may stem from several factors. First, variations in sample characteristics, such as age, cultural background, and occupation, can influence the manifestation of extraversion and its correlational pattern with creativity. Second, differences in creativity measurement tools may lead to divergent results; the present study adopted a trait creativity scale, and discrepancies in measured dimensions could account for the inconsistent findings. In this study, bivariate correlation analysis was used to examine the relationship between the modularity index (Q) and extraversion. The results are consistent with those of Kabbara et al. (2020) on extraversion and brain network functional connectivity, indicating that individuals with higher extraversion tend to have a higher modularity index (Q); in particular, functional connections are stronger in regions including the left paracentral lobule extending to the inferior parietal lobule, right lingual gyrus, and right supplementary motor area (Q. Li et al., 2025). One possible explanation is that higher extraversion may be associated with greater social engagement and environmental exploration, which could contribute to increased brain modularity and, in turn, higher creativity. However, since social activity and environmental exploration were not directly measured here, this interpretation remains hypothetical (Dumas et al., 2010; Tompson et al., 2020). We further conducted bivariate correlation analysis between the modularity index (Q) and trait creativity. The results are similar to previous research on trait creativity (Adnan et al., 2019; Beaty et al., 2015; Jiao et al., 2017; Kenett et al., 2020). This study found that individuals with higher modularity indices (Q) exhibited better trait creativity levels. High trait creativity may be associated with an efficient configuration of brain networks, where generating new ideas might rely on specific, efficient sub-networks; whereas reconstructing problem representations may require broader coordination across brain regions, primarily manifested as synchronized activation of more regions. This dynamic balance may be related to an optimized modular structure (Wu & Chen, 2023).

Mediation analysis results are consistent with a statistical mediation model in which the association between extraversion and trait creativity is statistically accounted for by the brain modularity index (Q). Previous work indicates that brain networks with higher modularity indices tend to be associated with more efficient resource allocation and linked to greater cooperative functioning across brain regions (Chaddock-Heyman et al., 2020). For instance, during divergent thinking tasks, higher modularity indices are related to more flexible switching of brain networks, which is associated with the generation of more novel ideas. In tasks involving insight, higher modularity may be linked to improved integration across brain regions and associated with restructured problem representation (Wu & Chen, 2023). These patterns are consistent with the view that a higher modularity index reflects stronger global brain coordination and functional integration capacity (Stevens et al., 2012). Specifically, extraversion scores are significantly and positively correlated with the strength of dynamic functional connections among the default mode network (DMN), salience network (SN), and executive control network (ECN) (Zou et al., 2018). Enhanced functional connectivity between the DMN and ECN enables individuals to better coordinate spontaneous thought and logical evaluation, thereby generating creative ideas that are both novel and practical (Shaw & Yu, 2023).

Therefore, a high modularity index is considered a beneficial characteristic for enhancing trait creativity. For individuals with higher extraversion, increased social activities and environmental exploration may enhance the modularity index of their brain networks (Tardiff et al., 2021), which in turn is positively associated with the enhancement of trait creativity. In summary, the present findings are consistent with a statistical mediation model in which the modularity index serves as a key mediator in the association between extraversion and trait creativity. These results support a statistical pathway in which the link between extraversion and trait creativity is statistically accounted for by individual differences in the brain network modularity index (Q). Furthermore, identification of this statistical mediating pattern provides important theoretical and empirical implications for future work exploring strategies that may be associated with enhanced creativity, such as neurofeedback training.

This study confirmed the statistical mediating role of the modularity index in the relationship between extraversion and trait creativity through resting-state brain network analysis, but several limitations warrant further discussion. First, this study employed a cross-sectional design, which precludes establishing causality. The proposed mediation model represents a statistical test based on theoretical assumptions rather than proof of a causal chain. All results should be interpreted as associations rather than causal relationships. Additionally, we used resting-state data to analyze the effect of extraversion on trait creativity, which captures only the average functional connectivity pattern over the entire scan, neglecting dynamic changes in brain networks. Future research could employ dynamic functional connectivity (dFC) approaches to explore how extraversion and trait creativity relate to the temporal flexibility of brain networks (Chen et al., 2025; Kabbara et al., 2020). Caution is warranted when interpreting this mediation model; our results indicate that the modularity index plays a statistically significant mediating role between these variables, but the practical effect size may be modest and could vary across different samples. The sample size (N = 43) is relatively small, which may lead to unstable parameter estimates and limited statistical power for mediation analysis. Additionally, all participants were university students, resulting in high homogeneity in age, education, and sociocultural background. These factors restrict the generalizability of our findings, as personality, creativity, and brain network organization vary substantially across age groups, educational levels, and social contexts. As an individual-difference study, future work should recruit larger and more diverse samples to validate the observed pathway. Furthermore, although our mediation model posited extraversion as the independent variable based on theory, other possibilities cannot be excluded, such as high creativity leading individuals to exhibit more extraverted behaviors, or a common neural basis simultaneously influencing both personality and creativity. Future longitudinal or interventional studies are needed to clarify these directions. Fourth, this study only measured extraversion and did not assess other factors that influence trait creativity, such as openness to experience and intelligence. Consequently, the unique contribution of extraversion to creativity and whole-brain modularity cannot be fully isolated. Future research should incorporate multiple personality dimensions and cognitive factors to clarify their relative contributions to trait creativity and whole-brain functional network architecture.

## 5. Conclusions

This study demonstrates a cross-sectional statistical pattern in which whole-brain functional network modularity is associated with extraversion and trait creativity, and shows a preliminary statistical mediation effect. Higher extraversion is associated with greater modularity, which in turn is associated with higher trait creativity. These findings provide preliminary associative evidence for a brain network basis linking personality to creative potential. All results should be considered preliminary and require replication in larger, more diverse samples with longitudinal designs and comprehensive covariate control.

## Figures and Tables

**Table 1:** Skewness and kurtosis of the data.

	Skewness	Kurtosis
Extraversion	−0.3510	2.4226
WCS	−0.2268	2.4165
Modularity (Q)	−0.1594	3.0335

Note: A skewness value close to 0 indicates a symmetric distribution; a kurtosis value close to 3 indicates a normal distribution (raw kurtosis).

**Table 2:** Correlations.

	*M*	*SD*	Extraversion	WCS	Modularity (Q)
Extraversion	56.51	10.27	-	0.38 *	0.37 *
WCS	111.12	11.57	0.38 *	-	0.41 **
Modularity (Q)	0.27	0.04	0.37 *	0.41 **	-

* *p* < 0.05. ** *p* < 0.01.

**Table 3:** Mediation analysis results (N = 43).

Model	Coeff	se	t	*p*	95% Bootstrap	
					LLCI	ULCI
a	0.371	0.145	2.557	0.014	0.086	0.711
b	0.305	0.15	2.036	0.048	0.042	0.583
c	0.384	0.144	2.659	0.011	0.089	0.703
c′	0.271	0.15	1.807	0.078	−0.047	0.605
a × b	0.113	\	\	0.111	0.003	0.290

## Data Availability

This study was conducted under the supervision of the College of Psychology, Liaoning Normal University. Due to data confidentiality requirements and ethical restrictions to protect research participants’ privacy, the data supporting the findings of this study are not publicly available. However, interested researchers may request access to the data after obtaining the necessary approval from the relevant authorities. All data requests should be directed to the corresponding authors of this study.

## Supplementary Materials

The following supporting information can be downloaded at: https://www.mdpi.com/article/10.3390/jintelligence14060094/s1, Table S1: Mediation Analysis Results of Neuroticism; Table S2: Mediation Analysis Results of Psychoticism.

## Abbreviations

The following abbreviations are used in this manuscript:

EPQ	The Eysenck Personality Questionnaire
Q	The modularity index of the whole brain
WCS	Williams Trait Creativity Tendency Inventory
