# Supplementary material for: Neural Correlates of Extraversion and Trait Creativity: A Graph Theory-Based Whole-Brain Functional Network Modularity Analysis
Source: J Intell. 2026 Jun 1;14(6):94. doi: 10.3390/jintelligence14060094 (PMC13302527; doi:10.3390/jintelligence14060094)
Supplement: Supplementary file 1 [file jintelligence-14-00094-s001.zip › jintelligence-4263689-supplementary.pdf]

For the sake of analytical completeness and to avoid selective reporting bias, we conducted the same analyses on the neuroticism and psychoticism dimensions of the EPQ-RSC. Firstly, raw scale scores were directly obtained and then converted into standard scores. Finally, to ensure statistical consistency, all scores were further transformed into standardized Z-scores. Subsequently, PROCESS Model 4 was adopted, and Bootstrap resampling with 5000 iterations was performed for correction. The results are presented in Table S1 and Table S2.

Table S1. Mediation Analysis Results of Neuroticism

					95% Bootstrap	
model	coeff	se	t	p	LLCI	ULCI
a	0.219	0.152	1.44	0.159	-0.043	0.457
b	0.4	0.148	2.7	0.01	0.145	0.673
c	0.109	0.155	0.703	0.486	-0.208	0.382
c'	0.022	0.148	0.146	0.885	-0.278	0.28
a*b	0.088			0.204	-0.013	0.233

Table S2.Mediation Analysis Results of Psychoticism

					95% Bootstrap	
model	coeff	se	t	p	LLCI	ULCI
a	0	0.156	0.003	0.996	-0.376	0.342
b	0.405	0.143	2.817	0.007	0.158	0.688
c	-0.092	0.156	-0.594	0.556	-0.341	0.17
c'	-0.0927	0.144	-0.644	0.523	-0.314	0.142
a*b	0			0.997	-0.149	0.173

The supplementary analyses showed that, in the mediation models for the neuroticism and psychoticism dimensions, the paths from the independent variable to the mediator (path "a") were both non-significant, and the 95% confidence intervals of the indirect effects both included zero. These results indicated that modularity did not mediate the relationships between neuroticism/psychoticism and creativity. Such findings suggest that the mediation pattern observed for the extraversion dimension did not replicate across the other personality dimensions of the EPQ-RSC, supporting the specificity of this association between extraversion and creativity.
